# New Books

**Published:** 2007-02

Alternative Pathways in Science and Industry: Activism, Innovation, and the Environment in an Era of Globalization

David Hess

Cambridge, MA:MIT Press, 2007. 360 pp. ISBN: 0-262-08359-0, $62

An Introduction to Pollution Science

Roy M. Harrison, ed.

New York:Springer, 2006. 298 pp. ISBN: 0-85404-829-4, $49.95

Assessing the Human Health Risks of Trichloroethylene: Key Scientific Issues

Committee on Human Health Risks of Trichloroethylene, National Research Council

Washington, DC:National Academies Press, 2006. 448 pp. ISBN: 0-309-10283-9, $59.40

Barry Commoner and the Science of Survival

Michael Egan

Cambridge, MA:MIT Press, 2007. 320 pp. ISBN: 0-262-05086-2, $28

Business and Environmental Policy

Michael E. Kraft, Sheldon Kamieniecki, eds.

Cambridge, MA:MIT Press, 2007. 376 pp. ISBN: 0-262-11305-8, $62

Chemoinformatics: Theory, Practice, and Products

B.A. Bunin, J. Bajorath, B. Siesel, G. Morales

New York:Springer, 2007. 295 pp. ISBN: 1-4020-5000-3, $129

Clearing the Air: The Health and Economic Damages of Air Pollution in China

Mun S. Ho, Christ P. Nielsen, eds.

Cambridge, MA:MIT Press, 2007. 392 pp. ISBN: 0-262-08358-2, $50

Compendium of Chemical Warfare Agents

Steven L. Hoenig

New York:Springer, 2006. 275 pp. ISBN: 0-387-34626-0, $99

Degrees That Matter: Climate Change and the University

Ann Rappaport, Sarah Hammond Creighton

Cambridge, MA:MIT Press, 2007. 376 pp. ISBN: 0-262-68166-8, 424.95

Environmental Justice and the Rights of Unborn and Future Generations: Law, Environmental Harm and the Right to Health

Laura Westra

London:Earthscan, 2006. 352 pp. ISBN: 1-84407-366-1, $110

Evolutionary Bioinformatics

Donald R. Forsdyke

New York:Springer, 2006. 424 pp. ISBN: 0-387-33418-1, $59.95

Flagging Standards: Globalization and Environmental, Safety, and Labor Regulations at Sea

Elizabeth R. DeSombre

Cambridge, MA:MIT Press, 2006. 280 pp. ISBN: 0-262-04234-7, $60

Gene Transfer; Delivery and Expression of DNA and RNA, A Laboratory Manual

Theodore Friedmann, John Rossi, eds.

Woodbury, NY:Cold Spring Harbor Laboratory Press, 2007. 793 pp. ISBN: 0-87969-764-4, $250

How Everyday Products Make People Sick: Toxins at Home and in the Workplace

Paul D. Blanc

Berkeley:University of California Press, 2007. 385 pp. ISBN: 0-520-24881-6, $50

Mathematics for Ecology and Environmental Sciences

Yasuhiro Takeuchi, Yoh Iwasa, Kazunori Sato, eds.

New York:Springer, 2007. 183 pp. ISBN: 3-540-34427-6, $119

Methods of Microarray Data Analysis V

Patrick McConnell, Simon M. Lin, Patrick Hurban, eds.

New York:Springer, 2006. 176 pp. ISBN: 0-387-34568-X, $99

Microarray Technology and Cancer Gene Profiling

Simone Mocellin, ed.

New York:Springer, 2007. 178 pp. ISBN: 0-387-39977-1, $139

Reproductive Health and the Environment

P. Nicolopoulou-Stamati, L. Hens, C.V. Howard, eds.

New York:Springer, 2006. 389 pp. ISBN: 1-4020-4828-9, $179

The Atlas of Climate Change: Mapping the World’s Greatest Challenge

Kirstin Dow, Thomas E. Downing

London:Earthscan, 2006. 128 pp. ISBN: 1-84407-376-9, $19.95

The Great Lead Water Pipe Disaster

Werner Troesken

Cambridge, MA:MIT Press, 2006. 296 pp. ISBN: 0-262-20167-4, $29.95

The World’s Water 2006–2007

Peter H. Gleick, Heather Cooley

Washington, DC:Island Press, 2006. 388 pp. ISBN: 1-59726-106-8, $35

There Is No Such Thing as a Natural Disaster: Race, Class, and Katrina

Gregory Squires, Chester Hartman, eds.

New York:Routledge, 2006. 328 pp. ISBN: 0-415-95486-X, $100

Toxicants in Aqueous Ecosystems

T.R. Crompton

New York:Springer, 2007. 456 pp. ISBN: 3-540-35738-6, $189

Translational Control in Biology and Medicine

Michael B. Mathews, Nahum Sonenberg, John W.B. Hershey

Woodbury, NY:Cold Spring Harbor Laboratory Press, 2007. 934 pp. ISBN: 0-87969-767-9, $135

